# Environmental Filtering Drives Microbial Community Shifts and Functional Niche Differentiation of Fungi in Waterlogged and Dried Archeological Bamboo Slips

**DOI:** 10.3390/jof12010066

**Published:** 2026-01-14

**Authors:** Liwen Zhong, Weijun Li, Guoming Gao, Yu Wang, Cen Wang, Jiao Pan

**Affiliations:** 1 Key Laboratory of Archaeomaterials and Conservation, Ministry of Education, University of Science and Technology Beijing, Beijing 100083, China; zhongleven@outlook.com (L.Z.); m202411471@xs.ustb.edu.cn (G.G.); d202310766@xs.ustb.edu.cn (Y.W.); d202410811@xs.ustb.edu.cn (C.W.); 2 Institute for Cultural Heritage and History of Science & Technology, University of Science and Technology Beijing, Beijing 100083, China; 3 Changsha Jiandu Museum, Changsha 410002, China; liweijun0119@foxmail.com

**Keywords:** archeological bamboo slips, environmental filtering, microbial community, biodeterioration, cultural relic preservation, fungal niches

## Abstract

Changes in preservation conditions act as an important environmental filter driving shifts in microbial communities. However, the precise identities, functional traits, and ecological mechanisms of the dominant agents driving stage-specific deterioration remain insufficiently characterized. This study investigated microbial communities and dominant fungal degraders in waterlogged versus dried bamboo slips using amplicon sequencing, multivariate statistics, and microbial isolation. Results revealed compositionally distinct communities, with dried slips sharing only a small proportion of operational taxonomic units (OTUs) with waterlogged slips, while indicating the persistence of a subset of taxa across preservation states. A key discovery was the dominance of *Fonsecaea minima* (92% relative abundance) at the water-solid-air interface of partially submerged slips. Scanning electron microscopy (SEM) and pyrolysis-gas chromatography/mass spectrometry (Py-GC/MS) indicate that this fungus forms melanin-rich, biofilm-like surface structures, suggesting enhanced surface colonization and stress resistance. In contrast, the fungal community isolated from dried slips was characterized by *Apiospora saccharicola* associated with detectable xylanase activity. Meanwhile, the xerophilic species *Xerogeomyces pulvereus* dominated (99% relative abundance) the storage box environment. Together, these results demonstrate that preservation niches select for fungi with distinct functional traits, highlighting the importance of stage-specific preservation strategies that consider functional traits rather than taxonomic identity alone.

## 1. Introduction

From discovery to conservation and exhibition, cultural relics are frequently subject to diverse environmental changes [1,2,3]. Among these factors, water content is a critical determinant of both the physical integrity and chemical stability of cultural materials [1]. Waterlogged materials are defined as water-saturated substrates that persist under anoxic or anaerobic conditions [4,5]. Waterlogged cultural heritage objects, such as wood and bamboo, are commonly recovered from aquatic contexts, including marine environments and underground water systems. In tidal zones, waterlogged wooden artifacts are exposed to recurrent cycles of drying and rewetting during low and high tides [6]. Notably, even under controlled indoor conservation conditions, preserved bamboo slips may experience analogous fluctuations in moisture availability, posing comparable risks of subsequent biodeterioration.

Bamboo slips represent a typical example of organic cultural materials that may endure cyclic drying and waterlogging even under indoor conservation conditions. Beyond China, bamboo and wooden slips were also used on the ancient Korean Peninsula and in Japan. Notably, their widespread adoption in these regions occurred after the gradual abandonment of bamboo and wooden slips in China. Comparable wooden writing media have likewise been identified in ancient Europe, dating from the late first to early second century CE, corresponding to the Eastern Han period in China [7,8]. As one of the most important cultural carriers in ancient China, bamboo slips served as the primary writing material prior to the widespread use of paper. Their manufacture involved cutting bamboo culms into segments, slicing them into thin slips, trimming and polishing the surfaces, and applying mild heating to remove residual moisture and insect eggs. Characters were written in ink on the yellow inner surface, and the finished slips were bound into volumes using silk strings [7]. In China, bamboo slips are predominantly discovered in Hunan Province and surrounding regions. Abundant groundwater systems resulted in tombs and wells remaining persistently water-saturated, causing archeological bamboo slips to be preserved in a waterlogged state. Long-term burial under such conditions exposes bamboo slips to continuous groundwater erosion and microbial colonization, leading to severe degradation of cellulose and hemicellulose and a substantial reduction in mechanical strength [9]. To maintain a condition comparable to their pre-excavation state, most waterlogged archeological bamboo slips are immersed in water immediately after excavation, preventing rapid dehydration, cracking, and shrinkage. In conservation practice, the drying stage refers to a critical stabilization process following long-term waterlogging, aiming to gradually remove moisture from internal pores and the cellulose network through controlled methods such as solvent exchange, freeze-drying, and polyethylene glycol infiltration [10,11], while preserving the microstructural integrity of the material to the greatest extent possible. Consequently, waterlogged and dried bamboo slips represent two distinct preservation states associated with fundamentally different environmental and microbial challenges.

In waterlogged stage, the microbiomes colonizing cultural relics in archeological bamboo slip are often dominated by bacteria and actinomycetes, a pattern commonly associated with oxygen-limited conditions. For example, Zoumalou bamboo slips (Three Kingdoms period, 220–280 CE, Wu State) exhibited biofilm formation by *Paracoccus* sp. and “eroded spot” deterioration caused by bacteria like *Bacillus cereus*, resulting in surface discoloration and structural damage [12]. Similarly, Chengdu Laoguanshan Slips (Han Dynasty in China, 206 BCE–220 CE) were affected by diverse deteriorative bacteria (e.g., *Cupriavidus*, *Staphylococcus*) and actinomycetes (e.g., *Promicromonospora*, *Streptomyces*), with *Cupriavidus* and *Staphylococcus* detected at both Zoumalou and Laoguanshan sites [13,14]. During this critical drying stage, new conservation challenges arise. Although molds such as *Aspergillus* spp. may be present but suppressed under oxygen-limited waterlogged conditions, the high organic content and residual moisture of bamboo slips render them highly susceptible to fungal colonization once exposed to air [12]. Fungi have been identified as primary degraders of waterlogged wood during wet-dry cycles, while white-rot fungi are the most important threat to waterlogged wooden artifacts during low and high tide [6]. This indicates that a shift in dominant biodeterioration agents may occur once waterlogged materials are no longer submerged, likely driven by the action of aerobic microbes, including fungi, that replace of bacteria and actinomycetes. Nevertheless, comprehensive studies identifying dominant fungal taxa and characterizing microbial community differentiation between waterlogged and dried states remain limited.

**Figure 1:**
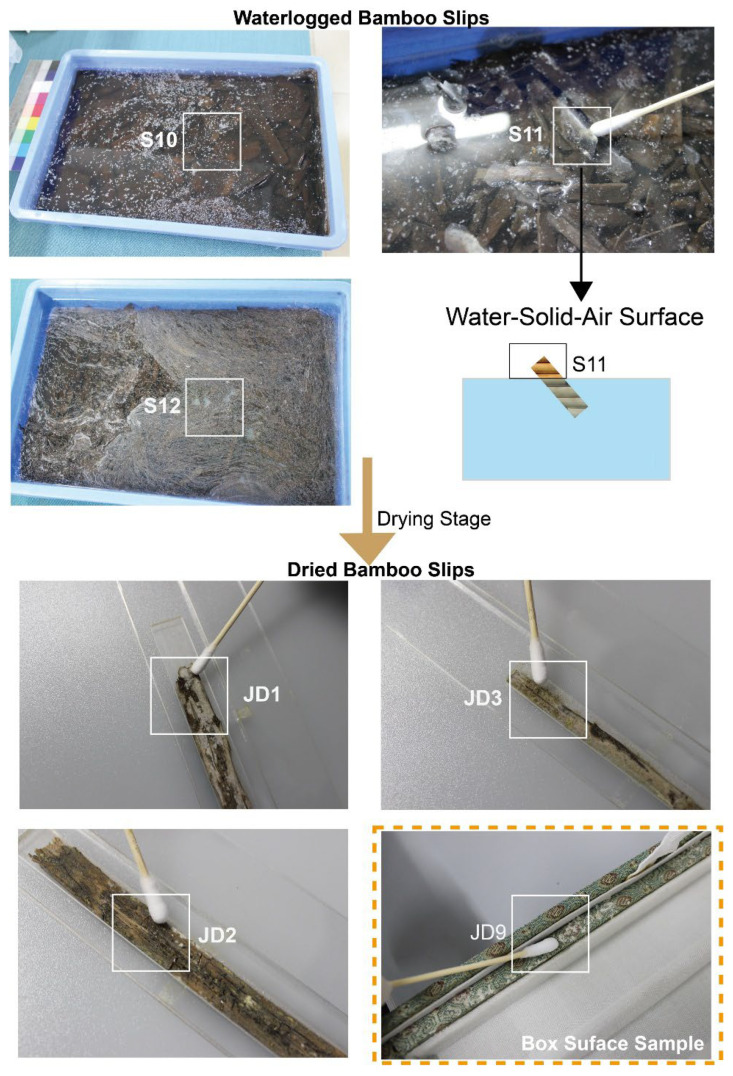
Sample collection of archeological bamboo slips. Samples included waterlogged slips (S10, S11, and S12), dried bamboo slip surfaces (JD1, JD2, and JD3) and the storage box surface sample (JD9).

In Zoumalou bamboo slips (Three Kingdoms period, Wu State) which were discovered in a well, some bamboo slips were stored in water, whereas others were carefully dried and preserved. Notably, we observed that the edges of some waterlogged bamboo slips were warped above the water surface, exposing themselves to air (Figure 1). This unique condition provides an ideal natural setting for studying fungal deterioration during occasional drying events. By combining fully waterlogged bamboo slips, dried bamboo slips, and this unique water-air-solid sample, we can conduct a comprehensive analysis of microbial deterioration throughout the process from waterlogged to drying and storage.

Accordingly, we collected waterlogged bamboo slips, including a unique sample exhibiting water-air-solid conditions, together with dried bamboo slips and storage box surface samples to investigate the microbial community compositions and fungal deterioration influenced by different preservation states (Figure 1). We characterized the microbial communities using amplicon sequencing and processed the data using multivariate statistical analysis to assess the *α* and *β* diversity across different preservation conditions. SEM observations, together with chemical evidence from Py-GC/MS, indicate that this fungus forms melanized, biofilm-like surface structures, which may contribute to surface colonization under environmental stress. The potentially important fungi colonizing on dried bamboo slips and box surface were isolated and identified. Collectively, these results demonstrate that distinct preservation habitats shape microbial community structure and drive niche-specific fungal biodegradation mechanisms in archeological bamboo slips.

## 2. Materials and Methods

### 2.1. Sampling of Microbial Communities in the Archeological Bamboo Slips

The Changsha Jiandu Museum (28°13′ N, 112°56′ E) is located at Changsha City, Hunan Province, China. These slips were excavated in 1996 from a well at the Zoumalou archeological site dating to Three Kingdoms period (220–280 CE) and have been preserved in the museum for approximately 30 years following their extraction (1996–2025). After extraction from the well (J22), all bamboo slips were washed with clean water. For most of the bamboo slips, the drying stage had been carried out by 2011, using a treatment based on ethanol and hexadecanol. In this method, ethanol was first used to replace water within the bamboo slips, followed by infiltration with a heated cetyl alcohol solution (60 °C), which displaced residual ethanol and reinforced the cellular structure, ultimately stabilizing the slips with minimal color or dimension change [15]. Since 2011, the dried bamboo slips have been sandwiched between acrylic sheets. For waterlogged bamboo slips, the soaking water is typically changed every two to three weeks. The specific interval depends on ambient temperature, with higher temperatures requiring more frequent water changes or adjustment when visible mold growth is observed. Ever since the completion of the drying stage, a portion of the bamboo slips have been stored in museum archival cases for approximately eight years. During sampling in October 2023, the ambient temperature was 18 °C and the relative humidity was maintained at 56%. Samples were collected from three distinct preservation stages, namely waterlogged bamboo slips, dried bamboo slips, and the storage box surface, yielding a total of seven samples (Figure 1). Specifically, from the waterlogged stage, three samples (S10, S11, and S12) were collected, comprising two fully submerged bamboo slips (S10 and S12) and one partially exposed slip at the water-air interface (S11). Additionally, three dried bamboo slips (JD1, JD2 and JD3) and one sample from the inner surface of storage box (JD9) were collected. Microbial colonies were sampled using sterile cotton swabs, which were then spread onto Potato Dextrose Agar (PDA) plates for the isolation of culturable fungi.

### 2.2. Scanning Electron Microscope (SEM) Observation

Microbial samples were mounted onto aluminum SEM stubs using carbon conductive adhesive. The samples were then dried in a desiccator. Gold sputtering was performed at a current of 24 mA for 300 s. Subsequently, the samples were examined using a SEM, and images were captured. The measurement parameters were set as follows: electron high tension (EHT) at 15.0 kV, working distance (WD) ranging from 9.5 to 10.7 mm, and magnification (Mag) between 2000× and 10,000×.

### 2.3. DNA Extraction, PCR Amplification and Sequencing of Isolated Fungi

Microbial colonies from waterlogged bamboo slips, dried bamboo slips, and box surface samples were inoculated onto PDA plates and incubated at 28 °C for 5–7 days until clear growth was observed. Strains were isolated by the streak plate method. A total of three fungal strains were isolated. Genomic DNA was extracted from fungal mycelia using a combination of glass bead disruption and phenol-chloroform extraction, as described by a previous study [16] with minor modifications. Briefly, the harvested mycelia were homogenized in lysis buffer with glass beads by vigorous vortexing. Nucleic acids were purified by phenol-chloroform extraction and precipitated with ethanol. The final DNA pellet was resuspended in nuclease-free water and used as a template for PCR. The internal transcribed spacer (ITS) region was amplified for all isolates (JDW11PF, JDD3LF, and JD9001) using primer pairs ITS1/ITS4 [17]. For isolate JDW11PF, additional markers, namely the partial sequence of the *β*-tubulin gene and the large subunit (LSU) rDNA were amplified using primers Beta 2a/Beta 2b and primers LROR/LR7 [18], respectively. The ITS region was amplified in a 25 μL PCR reaction mixture containing 12.5 μL of 2× GS Taq PCR Mix (Jinsha Biology, Beijing, China), 1 μL of each primer (10 μM), 40 ng of genomic DNA, and 8.5 μL of ddH_2_O. The PCR program was as follows: initial denaturation at 95 °C for 3 min; 30 cycles of denaturation at 94 °C for 25 s, annealing at 57 °C for 25 s, and extension at 72 °C for 30 s; followed by a final extension at 72 °C for 5 min. For isolate JDW11PF, the partial *β*-tubulin gene was amplified in a 20 μL PCR reaction mixture containing 10 μL of 2× GS Taq PCR Mix (Jinsha Biology, China), 1 μL of each primer (10 μM), 20 ng of genomic DNA, and 7 μL of ddH_2_O. The PCR condition was as follows: 95 °C for 3 min; 35 cycles of 94 °C for 25 s, 52 °C for 25 s, and 72 °C for 10 s; with a final extension at 72 °C for 5 min. The LSU rDNA region was amplified in a 25 μL PCR reaction mixture containing 12.5 μL of 2× GS Taq PCR Mix (Jinsha Biology, China), 1 μL of each primer (10 μM), 20 ng of genomic DNA, and 8.5 μL of ddH_2_O. The PCR program consisted of an initial denaturation at 95 °C for 3 min, followed by 35 cycles of 94 °C for 25 s, 57 °C for 25 s, and 72 °C for 15 s, with a final extension at 72 °C for 5 min. The amplified fragments were sequenced by GENEWIZ (Beijing, China).

### 2.4. Phylogenetic Identification of Screened Strains

A BLASTn search against the NCBI non-redundant nucleotide database (nt) (RID: PCMNNPEG014, accessed on 13 January 2025) in GenBank using the ITS region was carried out to determine the closest *Fonsecaea* taxa (JDW11PF, isolated from waterlogged bamboo slip S11). Additional sequences [19] were downloaded from GenBank. A maximum likelihood (ML) phylogenetic tree was reconstructed based on a concatenated dataset of the internal transcribed spacer (ITS), large subunit (LSU) ribosomal DNA, and *β*-tubulin gene sequences. Because only 19 reference strains with complete ITS, LSU, and *β*-tubulin sequences are currently available in NCBI, a single-gene tree based on ITS alone was used to supplement the concatenated analysis, which included a total of 52 sequences (Figure S2, Supporting Information). This strategy allowed a broader taxon sampling and improved phylogenetic resolution. For the analysis of strain JDD3LF (isolated from dried bamboo slip JD3), *Seiridium phylicae* was used as the outgroup. A maximum likelihood (ML) phylogenetic tree was constructed using a total of 50 sequences. The analysis was performed in accordance with the established taxonomic framework for the genus *Apiospora* [20,21]. According to the latest taxonomic revision [22], the genus *Xerogeomyces* is placed within the order Onygenales. Our target sequence (JD9001, isolated from box surface sample JD9) belongs to this genus, which is most closely related to genera such as *Dactylodendron*, *Emmonsiellopsis*, and *Sigleria*, with sequence similarities ranging from 88.64% to 92.58%. Accordingly, this framework was used as the basis to download ITS sequences of these genera and related taxa from NCBI for phylogenetic tree construction. Through this screening process, we ultimately obtained a high-quality dataset containing 14 ITS sequences. It should be noted that the total number of available sequences for the target clade (*Xerogeomyces* and its close relatives) in public databases remains relatively limited, highlighting that sequence data for this group remains limited. Sequence alignments were conducted using the ClustalW algorithm implemented in MEGA software (version 12.0). The best-fit substitution model was automatically selected by the software based on the Bayesian Information Criterion (BIC). The ML trees were inferred with 1000 bootstrap replicates to assess branch robustness.

### 2.5. DNA Extraction and Amplicon High-Throughput Sequencing of 16S and 18S rRNA Genes

Total genomic DNA was extracted using the DNeasy PowerSoil Pro Kit (QIAGEN, Hilden, Germany; Cat. No. 47014), strictly following the manufacturer’s protocols. The eukaryotic 18S rRNA gene V4 fragments were amplified using the primer pairs 528F (5′-GCGGTAATTCCAGCTCCAA-3′) and 706R (5′-AACTAAGACGGCCATGCA-3′). Prokaryotic 16S rRNA gene V4 regions were amplified using primers 515F (5′-GTGCCAGCMGCCGCGGTAA-3′) and 806R (5′-GGACTACHVGGGTWTCTAAT-3′) under the same PCR condition. All PCR reactions were carried out with 15 µL of Phusion^®^ High-Fidelity PCR Master Mix (New England Biolabs, Ipswich, MA, USA); 0.2 µM of forward and reverse primers, and about 10 ng template DNA. Thermal cycling consisted of initial denaturation at 98 °C for 1 min, followed by 30 cycles of denaturation at 98 °C for 10 s, annealing at 50 °C for 30 s, and elongation at 72 °C for 30 s, followed by final extension at 72 °C for 5 min. PCR products were purified using magnetic bead purification. Equimolar mixtures were electrophoresed on 2% agarose gels to recover target bands. Libraries were constructed, quantified by Qubit and Q-PCR, and sequenced on Illumina NovaSeq6000 (PE250 strategy). A total of 350,997 high-quality bacterial 16S rRNA sequences (Effective Tags) were obtained after chimera filtering and subsequently used for downstream ASV denoising (QIIME2-DADA2) and microbial community diversity analysis; 603,255 high-quality eukaryotic sequences were similarly processed. The raw sequencing data is publicly available in the NCBI Sequence Read Archive (SRA) under the study accession numbers PRJNA1357326 (18S) and PRJNA1357330 (16S).

### 2.6. Physiological Assays of Screened Fungi

The spore suspension (10^6^ spores) of fungal strains was added to a 250 mL Erlenmeyer flask containing 50 mL of medium composed of 0.5% (*w*/*v*) corn spelt xylan, 0.1% yeast extract, 0.07% K_2_HPO_4_, 0.02% KH_2_PO_4_, 0.1% (NH_4_)_2_SO_4_, and 0.11% MgSO_4_·7H_2_O (pH 7.0) [23]. The strain was cultured on a rotary shaking incubator at 180 rpm and 28 °C. The supernatant was collected as the crude enzyme extract for the assay of xylanase activity. The xylanase activities were assayed by 1% (*w*/*v*) of oat spelt xylan as substrate in a 100 mM sodium phosphate buffer (pH 7.0). The enzyme was incubated at 50 °C for 10 min. The amount of reducing sugars released during the reaction was quantified using the 3,5-dinitrosalicylic acid (DNS) method, with xylose used as the standard. One unit (U) of enzyme activity was defined as the amount of enzyme that releases 1 μmol of reducing sugar (xylose equivalent) per minute under the assay conditions.

### 2.7. Melanin Extraction

The extraction and purification processes were adapted from the literature [24]. *F. minima* was cultivated in 500 mL Erlenmeyer flasks containing 200 mL of Potato Dextrose Broth (PDB) at 28 °C and 180 rpm for 7 days, until the culture supernatant turned black. The mycelia from three parallel cultures (total volume ≈ 600 mL) were removed by vacuum filtration, and the resulting supernatant was collected as the melanin-containing fraction. The combined filtrates were acidified to pH 2.0 with 6 M HCl and allowed to precipitate overnight. The crude melanin was collected by gravity settling (room temperature, overnight) and washed 3–5 times with deionized water. The pellet was resuspended in 6 M HCl adjusted to pH 2.0 and heated at 100 °C for 2 h to hydrolyze residual carbohydrates and proteins. After hydrolysis, the suspension was cooled and collected by gravity settling (room temperature, overnight). The resulting precipitate was sequentially washed with organic solvents to remove lipids: first with chloroform, then with ethyl acetate, and finally with absolute ethanol. Each washing step was performed by vortexing the pellet with an equal volume of solvent, followed by air-drying at room temperature to allow complete solvent evaporation. The organic solvent washing cycle was repeated twice. The final melanin was air-dried at room temperature for about 12 h to obtain a black powder. Approximately 7 mg of melanin was obtained from the 600 mL of starting culture supernatant.

### 2.8. Fourier-Transform Infrared Spectroscopy (FT-IR)

The melanin extracted from *F. minima* was finely ground. The melanin powder was mixed with KBr (spectrally pure, Macklin, Shanghai, China) and pressed into pellets. The chemical structure of the samples was characterized using Fourier-transform infrared spectroscopy (FT-IR) (Nicolet™ iS™5, Thermo Scientific, Waltham, MA, USA). The absorption spectra were recorded in the range of 4000–800 cm^−1^, with baseline corrections performed at 4000, 1890, 1530, and 864 cm^−1^ [24].

### 2.9. Thermal Assisted Hydrolysis and Methylation Pyrolysis Coupled with Gas Chromatography/Mass Spectrometry (THM-Py-GC/MS)

THM-Py-GC/MS was employed to analyze the melanin extracted from *F. minima*. The analysis was performed using a Frontier Lab EGA/PY-3030D pyrolyzer (Frontier Laboratories Ltd., Koriyama, Fukushima, Japan) coupled to a Shimadzu GC/MS-QP2010 (Shimadzu Corporation, Takamatsu, Kagawa, Japan) Ultra system. Separation was achieved using a UA-5MS capillary column (30 m × 0.25 mm i.d., 0.25 µm film thickness). The pyrolysis was conducted at 770 °C for 8 s, with the GC injector temperature set at 320 °C in a split mode (split ratio 30:1). The carrier gas was helium at a constant flow rate of 1.0 mL/min. The GC oven temperature was programmed as follows: initial temperature of 40 °C (held for 1 min), ramped to 80 °C at 5 °C/min, then to 280 °C at 15 °C/min, and finally held at 280 °C for 15 min. The mass spectrometer was operated in the electron ionization (EI) mode at 70 eV. The ion source temperature was 230 °C, and the transfer line temperature was 300 °C. The mass spectra were acquired in full scan mode over a mass range of *m*/*z* 30–550 [25].

## 3. Results

### 3.1. Microstructural Observation of Microorganisms on Waterlogged, Dried Bamboo Slips and Box Surface via Scanning Electron Microscopy

**Figure 2:**
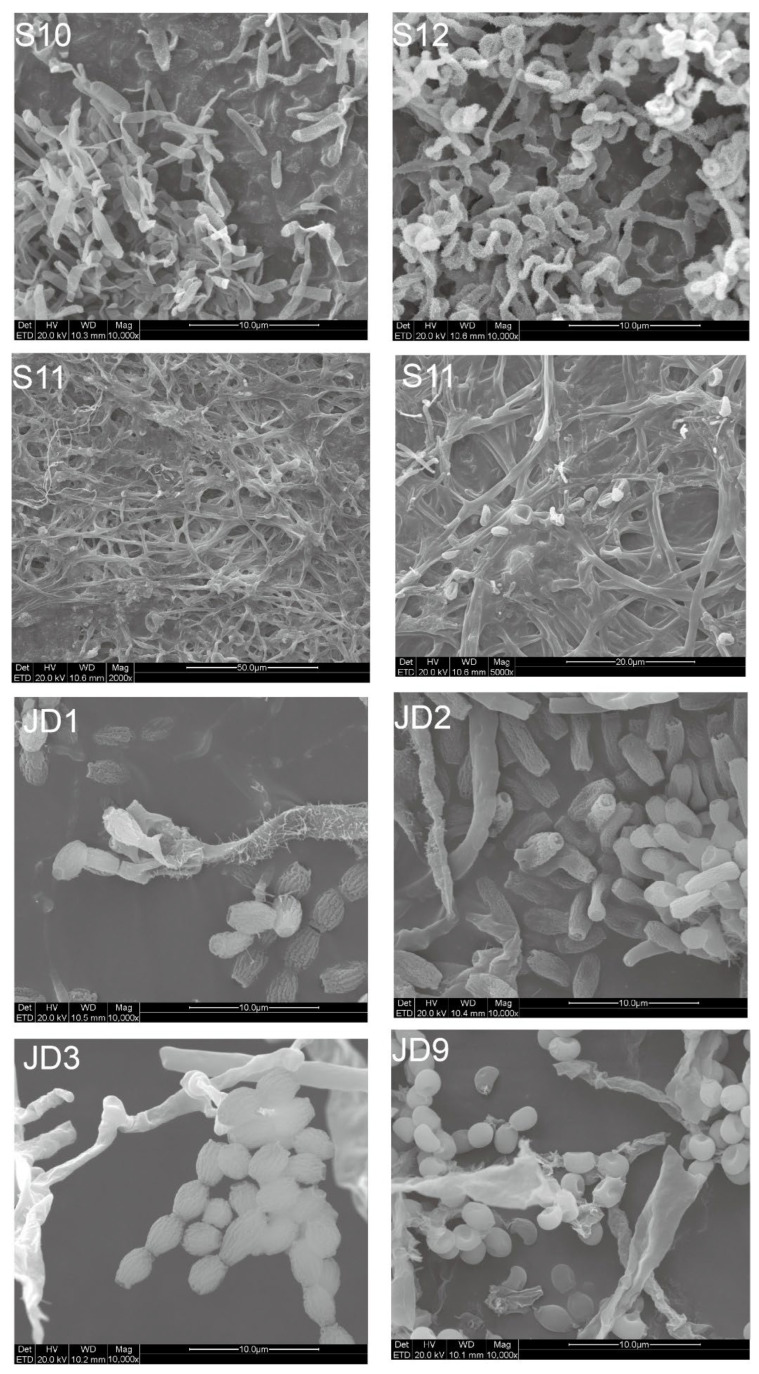
Photomicrographs of colonies on archeological bamboo slips at different preservation stages as revealed by scanning electron microscopy (SEM). Waterlogged bamboo slips (S10–S12), while S11 at water-solid-air surface. JD1–3 on dried bamboo slips and JD9 on the box surface with microbial plaque.

Scanning electron microscopy (SEM) observations revealed a significant number of discretely distributed vesicular-like structure in the S10 sample (Figure 2, sample S10). These structures appeared as independent, approximately ellipsoidal sac-like forms, with diameters ranging from 0.5 to 2 μm. The external surfaces were rough and densely covered with spiny protrusions. For the other waterlogged bamboo slip sample S12 (Figure 2, sample S12), helical structures were observed, with diameters of approximately 0.5–1 μm, and their surfaces also exhibited spiny protrusions. In the water-solid-air sample S11 (Figure 2, sample S11), fungal hyphae were observed forming densely intertwined filamentous structures, which may suggest a three-dimensional hyphal organization, although preparation-related artifacts cannot be fully excluded (Figure S1). The hyphae exhibited diameters of 1–3 μm.

On the dried bamboo slips (Figure 2, sample JD1, JD2, and JD3), the observed microbial structures differed markedly from those on the waterlogged samples. On the surface of the dried bamboo slip JD1, numerous spore-like structures were scattered across, measuring approximately 4–5 μm in diameter and ~4 μm in length, with flattened ends and a wrinkled surface. Hyphae-like structures with diameters of approximately 1–3 μm were also present. On JD2, capitate structures surrounded by dispersed spores were observed. The spores measured approximately 2–2.5 μm in diameter and 3–5 μm in length, with a wrinkled surface. On JD3, spores morphologically identical to those observed on JD1 were present.

On the box surface sample JD9 (Figure 2, sample JD9), which exhibited visible microbial accumulation, scattered circular spores with a central depression were observed. The spores measured approximately 1–2 μm in diameter and exhibited smooth surfaces.

### 3.2. Diversities of the Microbial Communities of Archeological Bamboo Slips in Different Conservation Stages

**Figure 3:**
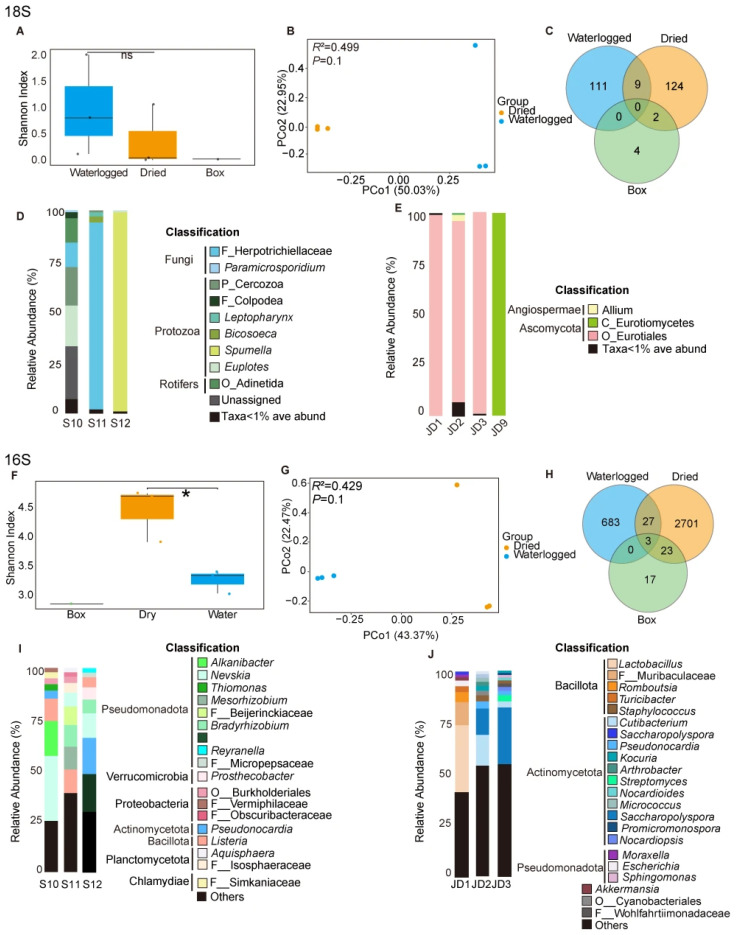
Microbial community structure of dried and waterlogged bamboo slips and the box surface sample. (**A**) Boxplot showing the Shannon indices of eukaryotic communities across the three groups (Student’s *t*-test: ns, not significant). (**B**) PCoA scatter plot representing the *β* diversity of eukaryotic communities across the three groups, with PERMANOVA *R*^2^ and *p* values indicated. (**C**) Venn diagram illustrating the distribution of eukaryotic OTUs across waterlogged slips, dried slips, and the box surface sample. (**D**,**E**) Relative abundance of the eukaryotic community in waterlogged bamboo slips (**D**) and in dried slips and the box surface sample (**E**). (**F**) Boxplot showing the Shannon indices of prokaryotic communities across the three groups (*: *p* < 0.05). (**G**) PCoA scatter plot representing the *β* diversity of prokaryotic communities across the three groups, with PERMANOVA *R*^2^ and *p* values indicated. (**H**) Venn diagram illustrating the distribution of prokaryotic OTUs across waterlogged slips, dried slips, and on the box surface sample. (**I**,**J**) Relative abundance of the prokaryotic community in waterlogged bamboo slips (**I**) and in dried slips and the box surface sample (**J**). All bacterial OTUs in JD9 are below 1% abundance and are not labeled in panel J. In the taxonomic classification, G, F, and O refer to genus, family, and order, respectively. Detailed sample information is provided in Figure 1.

The Shannon index, which reflects both the richness and evenness of the microbial composition, was used to describe *α* diversity across waterlogged bamboo slips, dried bamboo slips and box surface samples. Based on the 18S rRNA gene data (Figure 3A), no statistically significant difference in eukaryotic *α* diversity was detected between waterlogged and dried bamboo slips (*t* = 0.93, *p* = 0.41), although the waterlogged group (1.03 ± 0.96) exhibited a numerically higher Shannon index than the dried group (0.42 ± 0.60). Since the box group contained only one sample (JD9), statistical testing was not performed, and its Shannon index value (0.06838) is reported descriptively. In contrast, analysis of the 16S rRNA gene data revealed a significant difference in prokaryotic α diversity between waterlogged and dried samples (*t* = 4.09, *p* = 0.033), with the dried group (4.51 ± 0.47) showing a significantly higher Shannon index than the waterlogged group (3.32 ± 0.20) (Figure 3F). The box surface sample (JD9), had a Shannon index of 2.91 and was excluded from statistical testing due to the lack of replication.

The *β* diversity of the eukaryotic communities was described by PCoA based on Bray−Curtis dissimilarities. No significant clustering was observed between waterlogged and dried bamboo slips (PERMANOVA 18S rDNA, *R*^2^ = 0.4988, *p* = 0.1; ANOSIM: *R* = 1, *p* = 0.1, Figure 3B). Similarly, prokaryotic communities did not exhibit statistically significant separation between the two preservation groups (PERMANOVA 16S rDNA, *R*^2^ = 0.4286, *p* = 0.1; ANOSIM: *R* = 1, *p* = 0.1, Figure 3G). The box surface sample (JD9) was excluded from PCoA analysis due to the presence of only one replicate. The lack of significant findings for both the eukaryotic and prokaryotic communities may be due to the small sample size.

Venn diagrams of both 16S and 18S rRNA genes datasets revealed pronounced differences in community composition among preservation stages. For eukaryotes (18S) (Figure 3C), the dried slips showed the highest unique diversity (49.6% OTUs), compared to the waterlogged slips (44.4% OTUs) and the box surface (1.6% OTUs). In contrast, for prokaryotes (16S) (Figure 3H), the dried bamboo slips hosted the highest diversity (78.2% OTUs), vastly outnumbering the waterlogged slips (19.8% OTUs) and the box surface (0.5% OTUs). Only a small fraction of OTUs was shared among the three groups (4.4% for 18S, 1.5% for 16S).

In summary, the microbial communities on the bamboo slips underwent pronounced compositional changes following the drying stage. The prokaryotic communities (bacteria/archaea) exhibited a significant increase in *α* diversity after the drying stage, whereas the eukaryotic *α* diversity remained comparatively stable. Although *β* diversity analyses did not reveal statistically significant separation, the limited overlap of OTUs highlights strong stage-specific microbial assemblages.

### 3.3. High-Throughput Sequencing of Microbial Communities in Different Preservation Stages and Dominant Isolated Fungi

Across the waterlogged archeological bamboo slips (S10, S11, and S12), 18S rRNA gene amplicon sequencing revealed distinct eukaryotic community structures. In sample S10, the eukaryotic community comprised *Euplotes* (20% at the genus level), Cercozoa (19% at the phylum level), Adinetida (12% at the order level), and Herpotrichiellaceae (12% at the family level). Herpotrichiellaceae at family level was highly predominant in S11 (92%), while *Spumella* at genus level was overwhelmingly dominant in S12 (98%). There are three major groups of eukaryotic communities in the waterlogged samples, namely fungi, protozoa and rotifers. Both S10 and S12 were dominant by protozoa and rotifers (66% in S10 and 99% in S12), while S11 was dominant by fungi (92%).

**Figure 4:**
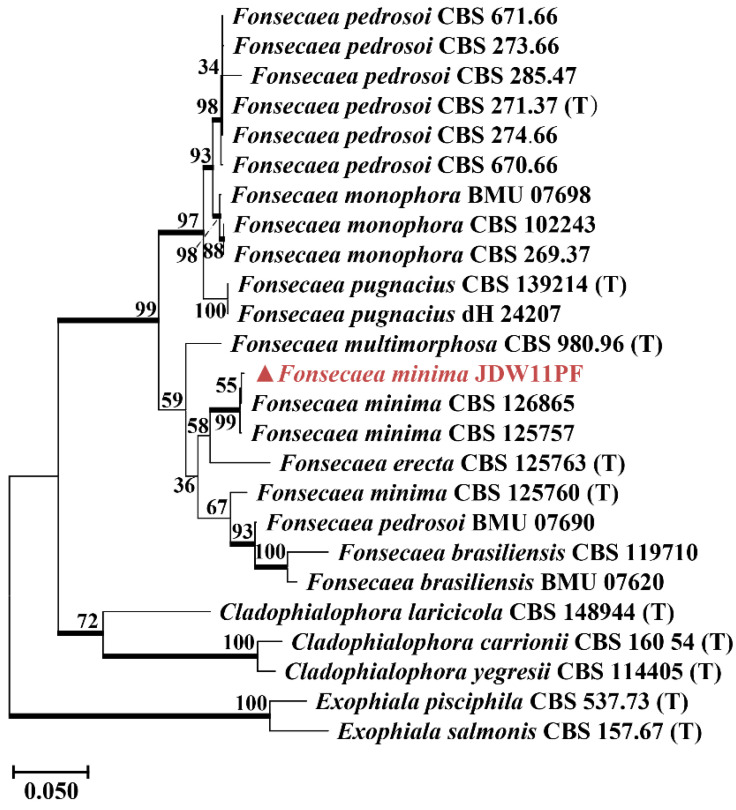
Maximum likelihood tree of *F. minima* JDW11PF based on concatenated sequences of the ITS, LSU, and *β*-tubulin genes. *Cladophialophora laricicola*, *Cladophialophora carrionii*, *Cladophialophora yegresii*, *Exophiala salmonis*, and *Exophiala pisciphila* were used as the outgroup. The strain *F. minima* JDW11PF isolated from sample S11, clustered within the *F. minima* clade with high support (99%). (T) = type strain of the species. Bootstrap support was calculated from 1000 replicates; values > 70% were indicated in bold on the branches. In the phylogenetic tree, the strain isolated from sample S11 in this study is highlighted in red, and a triangle is used to mark all fungal strains isolated in this study.

Cultivation-based isolation yielded only one fungal taxon that aligned with these high-abundance sequences in the S11 sample with Herpotrichiellaceae (92% at the family level). The isolated fungus was identified as *Fonsecaea minima* (Figure 3D, Figure 4 and Figure S2). Since *F. minima* belongs to Herpotrichiellaceae, and given the limited taxonomic resolution of 18S rRNA gene sequencing, along with the consistency between its morphology and the original sample’s macroscopic appearance (Figures S1 and S2), the combined evidence supports that *F. minima* represents the dominant fungal taxon in S11. The inability to isolate *Euplotes* or *Spumella*, despite their high sequence abundance, reflects the well-recognized difficulty of cultivating many microeukaryotes under standard laboratory conditions.

**Figure 5:**
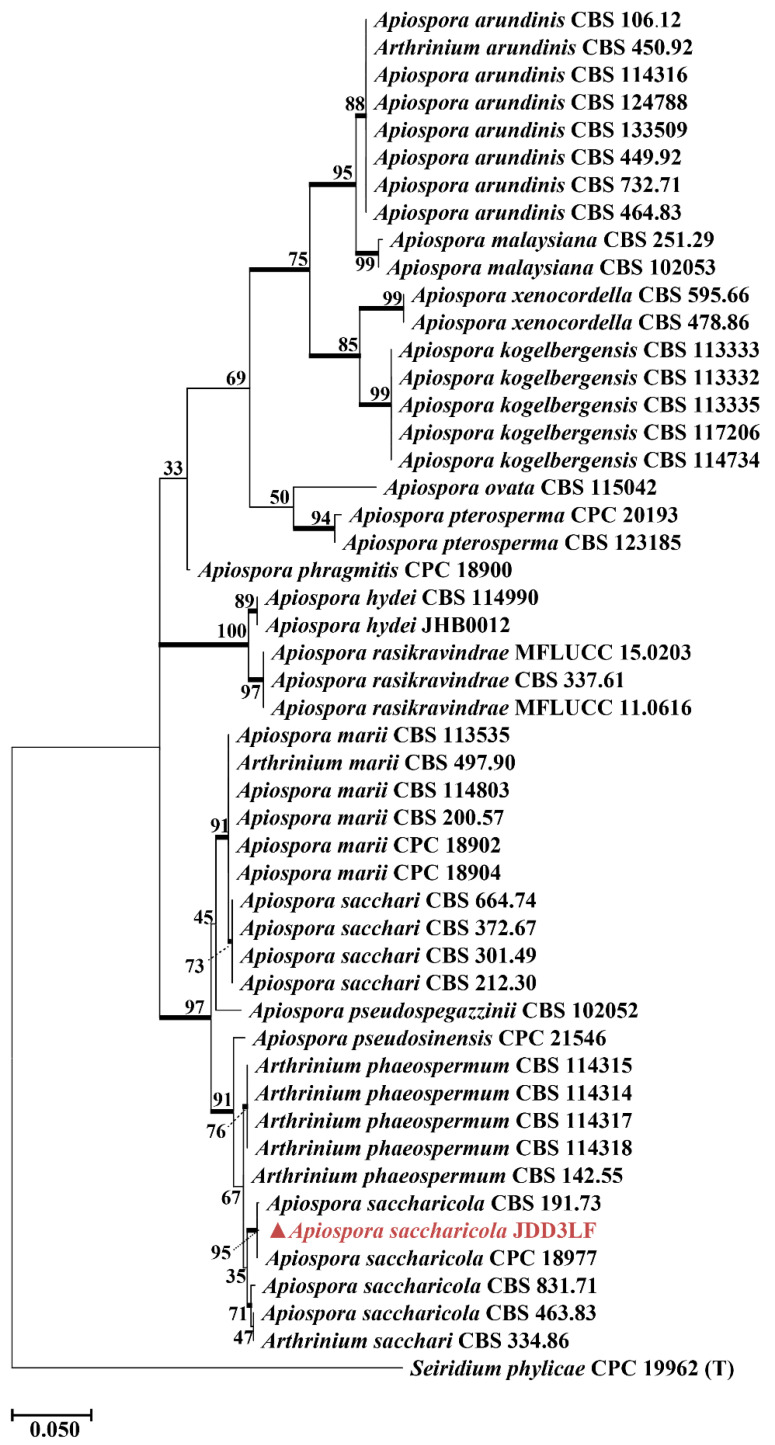
Maximum likelihood tree of *A. saccharicola* JDD3LF based on ITS genes. *Seiridium phylicae* was used as the outgroup. The strain *A. saccharicola* JDD3LF clustered with the *A. saccharicola* CBS 191.73 with high support (95%). The genus name *Apiospora* is used here, which encompasses species formerly classified under *Arthrinum* sensu lato. (T) = type strain of the species. Bootstrap support was calculated from 1000 replicates; values > 70% were indicated in bold on the branches. In the phylogenetic tree, the strain isolated from sample JD3 in this study is highlighted in red, and a triangle is used to mark all fungal strains isolated in this study.

**Figure 6:**
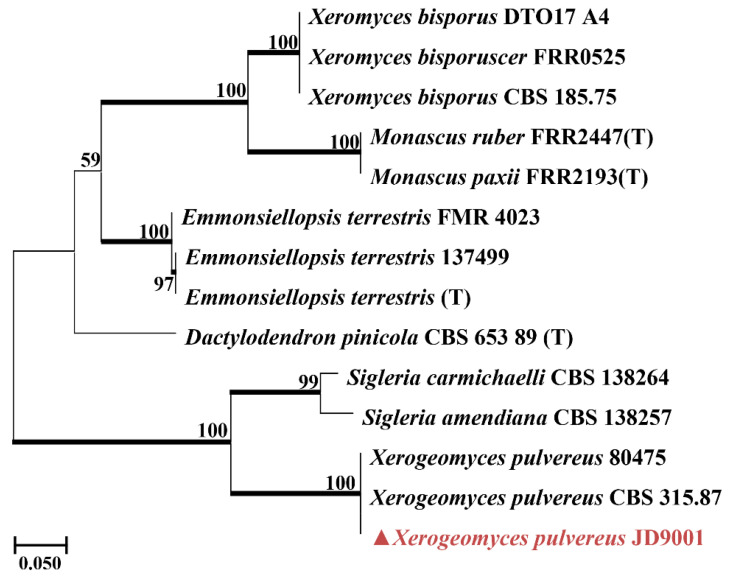
Maximum likelihood tree of *X. pulvereus* JD9001 based on ITS genes. *Monascus ruber*, *M. paxii*, *Xeromyces bisporus*, and *Xeromyces bisporuscer* were used as the outgroup. The newly sequenced strain *X. pulvereus* JD9001 (highlighted in red) formed a well-supported clade (100% bootstrap) with the type strain *X. pulvereus* CBS 315.87 (T), confirming its species identity. This clade, representing the monotypic genus *Xerogeomyces*, was recovered as a distinct lineage sister to the morphologically similar genera *Sigleria* and *Dactylodendron*, consistent with recent taxonomic revisions [22]. The broader clade containing these three genera was placed within the Onygenales, with *Emmonsiellopsis* as a more distantly related sister group. (T) = type strain of the species. Bootstrap support was calculated from 1000 replicates; values > 70% were indicated in bold on the branches. In the phylogenetic tree, the strain isolated from sample JD9 in this study is highlighted in red, and a triangle is used to mark all fungal strains isolated in this study.

Analysis of the dried bamboo slips revealed a marked methodological contrast. 18S rRNA gene amplicon sequencing revealed Eurotiales at the order level were dominant taxa (Figure 3E) in JD1 (99%), JD2 (89%) and JD3 (99%), which aligned with the results of TA-cloning of ITS regions (Figures S4 and S5), where 19/19 clones from JD1 and 20/20 clones from JD2 were assigned to the genus *Aspergillus*, as *Aspergillus* belonged to the order Eurotiales. Despite repeated attempts, *Aspergillus* was not successfully cultured. Instead, *Apiospora saccharicola*, a known biodegradation agent of bamboo materials, was readily isolated and identified (Figure 5), despite being undetectable (<0.1%) in both sequencing datasets. In contrast, *Xerogeomyces pulvereus* was successfully identified (Figure 6) as the dominant colonizer through a combination of TA-cloning (ITS region: 18/18 clones; Figure S6), 18S amplicon sequencing, cultivation, and macroscopic observation. This finding is consistent with the 18S data, which showed that Eurotiomycetes (the class to which *X. pulvereus* belongs) dominated (99%) the community.

Across the waterlogged (S10, S11, and S12) and dried archeological bamboo slips (JD1, JD2, and JD3), 16S rRNA gene amplicon sequencing revealed distinct prokaryotic community structures. For waterlogged samples (Figure 3I), at the genus level, the most abundant genus of S10 was *Nevskia* (25.3%). Sample S11 exhibited relatively even abundances among several genera, including *Listeria* (11.2%), *Mesorhizobium* (10.9%), and *Bradyrhizobium* (10.3%). The most abundant genus of S12 is *Pseudonocardia* (14.7%). At the phylum level, all three waterlogged samples shared the same abundant phylum Pseudomonadota (54.6% in S10, 50.7% in S11 and 34.3% in S12), and S12 has a member of Actinomycetota (14.7%). For dried bamboo samples (Figure 3J), at the genus level, the most abundant genus of JD1 was *Lactobacillus* (33.6%), the most abundant genera of JD2 were *Cutibacterium* (15.1%) and *Saccharopolyspora* (12.9%), while the most abundant genus of JD3 was *Saccharopolyspora* (26.1%). At phylum level, both 37.38% of JD2 and 38.7% of JD3 belonged to the Actinomycetota, while 41.3% of JD1 belonged to the Bacillota. All bacterial OTUs detected in JD9 were below 1% relative abundance, indicating the absence of a dominant prokaryotic population on the box surface.

**Figure 7:**
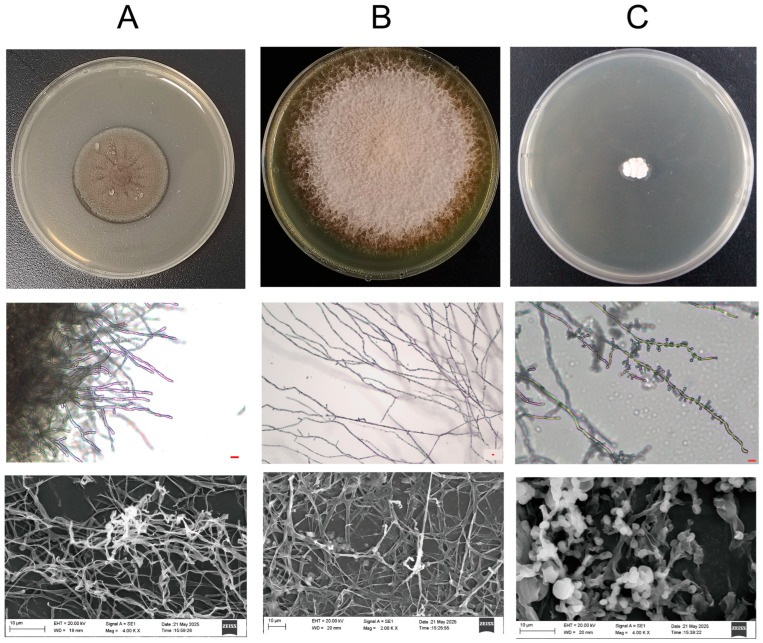
Macromorphologies on PDA medium, light micrographs, and scanning electron micrographs of the three fungal strains isolated from different preservation stages. (**A**) *F. minima* isolated from water-solid-air sample (S11). (**B**) *A. saccharicola* isolated from dried bamboo slips (JD3). (**C**) *X. pulvereus* isolated from the box surface (JD9). Scale bar: 10 μm (applicable to light micrographs).

In conclusion, the integration of molecular approaches (18S amplicon sequencing, ITS cloning, and 16S amplicon sequencing) with cultivation-based isolation revealed the complex microbial communities inhabiting the waterlogged (S10-S12) and dried archeological bamboo slips (JD1-JD3), as well as the storage environment (JD9). This synergy led to the isolation of three key fungal taxa: *F. minima*, *A. saccharicola*, and *X. pulvereus *(Figure 7). Notably, *A. saccharicola* and *F. minima* were isolated directly from the bamboo surfaces (JD3) and water-solid-air samples (S11), respectively, supporting their direct participantion in the biodegradation process. Furthermore, combined molecular and cultivation evidence consistently indicated that *X. pulvereus* was the dominant species on the box surface.

### 3.4. Melanin Production of F. minima Isolates

To investigate the physiological traits of *F. minima*, we conducted enzymatic activity assays and analyzed melanin production using FTIR and Py-GC/MS. However, no detectable enzymatic activity related to cellulase or hemicellulase production was observed in the plate assays.

Melanin is a macromolecule formed by oxidative polymerization of phenolic or indolic compounds. The generally accepted chemical criteria used to define melanin include the following: a black color, insolubility in both cold and boiling water as well as organic solvents, resistance to degradation by concentrated acids, resistance to bleaching by oxidizing agents such as hydrogen peroxide, and susceptibility to solubilization and degradation by hot alkali solutions. In fungi, several different types of melanin have been identified to date. The two most important types are DHN-melanin (named for one of the pathway intermediates, 1,8-dihydroxynaphthalene) and DOPA-melanin (named for one of the precursors, L-3,4- dihydroxy phenylalanine) [26,27].

**Figure 8:**
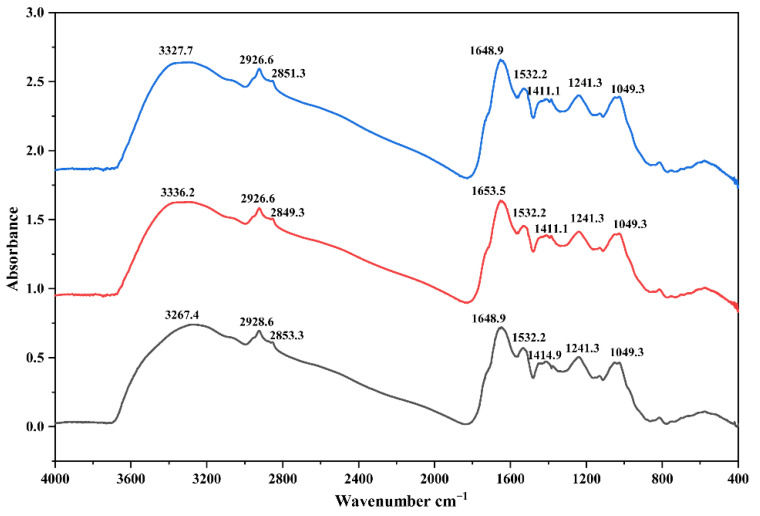
Fourier Transform Infrared Spectroscopy of Melanin Extracted from *F. minima*. The spectra of three technical replicates are shown in different colors (blue, red and black).

The extracted pigment was characterized by FTIR spectroscopy to identify its functional groups (Table 1, Figure 8). The spectrum exhibited characteristic absorption bands indicative of melanin-like compounds. A broad and intense band in the region of 3300–3500 cm^−1^ was observed, which can be assigned to the stretching vibrations of O-H or N-H groups [28,29,30]. Bands at 2926 cm^−1^ and 2850^−1^ can be assigned to the stretching vibrations of aliphatic C-H group (νC-H_2_, νC-H_3_) [28,30,31]. Bands at 1648–1653 cm^−1^ were attributed to conjugated C=O and C=C stretching vibrations in quinoid or aromatic structures [29,31,32]. A notable and well-defined absorption band was observed at 1532 cm^−1^, which is characteristic of N-H in-plane bending vibrations coupled with C-N stretching vibrations (secondary amines), often associated with indolic or amide functionalities [29,30,31,32]. Additional bands were identified at 1411–1415 cm^−1^ (C-N stretching and C-H deformations) [28,29,30,31,32], 1231–1239 cm^−1^ (C-OH stretching vibrations of phenolic, carboxylic, and alcoholic groups) [28,30,31,32], and 1049–1051 cm^−1^ [28,29,30,31,32] (C-O stretching of alcoholic groups and in-plane C-H deformations). The FTIR spectral features collectively indicate the presence of a nitrogen-containing aromatic polymer with functional groups characteristic of melanin-like pigments, thereby providing strong spectroscopic evidence for the existence of melanin-like components in the pigment. To further elucidate the specific structural units and confirm the presence of key building blocks such as pyrrole and indole, the pigment was subjected to Py-GC/MS.

**Figure 9:**
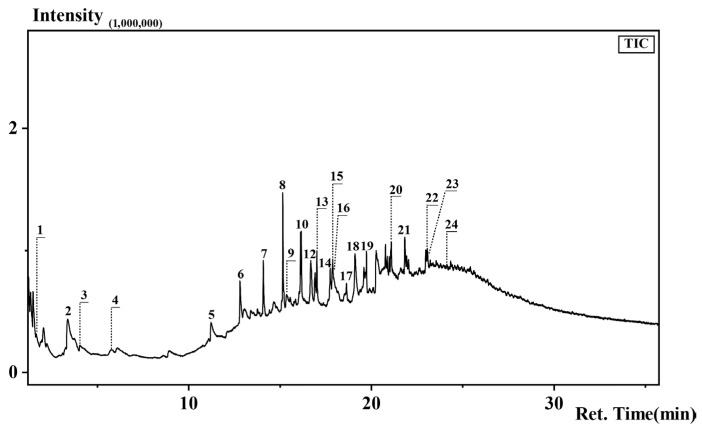
The Total Ion Chromatograms (TIC) of The Melanin Extracted from *F. minima*.

The Py-GC/MS profile revealed diagnostic markers of eumelanin, most significantly indole (Peak 9: 2.89%) (Table 2, Figure 9), which is commonly derived from the thermal degradation of 5,6-dihydroxyindole (DHI) units in DOPA melanin polymers. Complementary evidence was provided by the detection of pyrrole derivatives (Peak 3: 0.57%), consistent with the heterocyclic core structure of eumelanin. The absence of characteristic DHN-melanin pyrolysis products suggests that the polyketide-derived DHN pathway is unlikely to be involved [24].

Based on the integrated analytical results from FTIR and Py-GC/MS, the melanin extracted from *F. minima* was characterized as DOPA-type eumelanin, likely complexed with lipids and proteins, consistent with previous reports of fungal melanin composition (Figure 8 and Figure 9).

### 3.5. Xylanase Activity and Biomass Test

**Figure 10:**
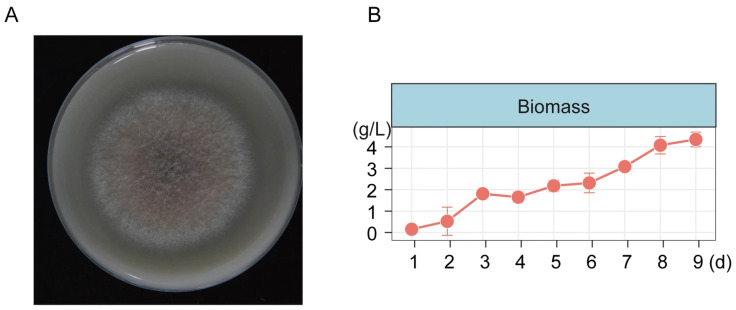
*A. saccharicola* Hemicellulose Detection Medium (**A**) and Fungal Growth Curve (**B**).

Enzyme activity analysis revealed that *A. saccharicola* exhibited xylanase activity on hemicellulose detection medium, forming a clear hydrolysis zone (Figure 10A). Furthermore, the xylanase activity in the culture supernatant was determined to be (3.38 ± 0.52) × 10^−4^ U/mL after 7 days of incubation.

## 4. Discussion

The transition of bamboo slips from a waterlogged to a dried state induces substantial changes in the physical properties of the material, which in turn drive shifts in associated microbial communities. Although *β* diversity analysis did not reveal statistically significant separation for either prokaryotic or eukaryotic communities (Figure 3B,G), the very low proportion of shared OTUs (4.4% for 18S, 1.5% for 16S), as observed in Venn diagrams (Figure 3C,H), indicates pronounced taxonomic turnover across stages in both domains. In parallel, compared with waterlogged bamboo slips and box surface samples, the dried bamboo slips exhibited a significantly higher prokaryotic *α* diversity (Figure 3F). These results suggest that the drying process selectively eliminated original aquatic microorganisms while facilitating the establishment and dominance of a more diverse assemblage of drought-tolerant taxa. This pattern is consistent with observations from other heritage contexts, such as the Dahuting Han Dynasty Tomb, where relatively stable microclimatic conditions functioned as an environmental filter, favoring specific dominant microbial taxa, including members of the Pseudonocardiaceae [3].

The principle of environmental filtering is also clearly reflected in the eukaryotic communities. The waterlogged slips were predominantly characterized by aquatic protists like *Euplotes* (20%) and *Spumella* (98%) [33,34,35]. In contrast, a single fungal taxon, Herpotrichiellaceae (Figure S3), overwhelmingly dominated (92%) a distinct sample located at the water-air-solid interface. This finding is of particular importance because the strain was successfully isolated. SEM observations of sample S11 showed densely distributed and intertwined hyphal structures forming a network-like morphology on the surface (Figure 2, sample S11), which suggests extensive fungal colonization at the water-solid-air interface. The greenish-black colonies of the isolate were consistent with the fungal plaque observed on the original sample (Figure S7), confirming *F. minima* as the dominant component of sample S11. Conversely, the dried slips were dominated by the filamentous fungus *Aspergillus* (95%), a genus well known for its capacity to colonize cellulosic substrates such as paper [36]. The dominance of *Aspergillus* spp. on dried bamboo slips was further corroborated by SEM observations and the consistency of TA-clone sequencing results (Figures S3 and S4), with the surfaces of the dried bamboo slips (Figure 2, sample JD1, JD2 and JD3) exhibiting highly similar spore and hyphal morphologies. Furthermore, the xerophilic fungus *X. pulvereus* isolated from the box surface was also demonstrated to be dominant. This was supported by the concordance between in situ observations of colonies on the original sample (Figure 2, sample JD9) and the pure-culture morphology of *X. pulvereus* (Figure 7C), as well as the identical depressed spore structures observed by SEM in both the isolate (Figure 7C) and the original sample (Figure 2, sample JD9), together with its high abundance revealed by TA-cloning sequencing (ITS region, 18/18 clones; Figure S5) and 18S amplicon sequencing (Figure 3E).

The prokaryotic community patterns further support the principle of environmental filter, as a subset of prokaryotic taxa was shared between waterlogged and dried bamboo slips. The prokaryotic communities in the waterlogged samples were mainly composed of Pseudomonadota and Actinomycetes, whereas the dried samples were dominated by Actinomycetes. The taxonomic composition of the waterlogged slips strongly reflects an aquatic environment that is highly susceptible to external inputs. For instance, the high abundance of *Nevskia* (25.3% in S10), a biofilm-forming genus commonly occurring at the water-air interface [37], is characteristic of such conditions. Furthermore, the microbial assemblage in S11, featuring potential zoonotic pathogens like *Listeria* (11.2%) and plant-symbiotic rhizobia such as *Mesorhizobium* (10.9%) and *Bradyrhizobium* (10.3%), suggests possible eutrophication [38,39]. Such external organic inputs likely generated a nutrient-enriched microenvironment. The detection of *Cutibacterium* (15.1% in JD2), a genus commonly associated with human skin, further indicates possible contamination during handling [40].

In addition to these distinctions, a clear continuity between waterlogged and dried bamboo slips was observed in terms of prokaryotic taxa persisting on the bamboo material. Notably, *Lactobacillus* exhibited a high relative abundance (33.6% in JD1) in dried bamboo slips, despite being facultative anaerobes [41]. This unexpected persistence suggests that these taxa originated from the preceding waterlogged, oxygen-limited conditions and survived the subsequent drying stage. These observations highlight the drying stage as a critical but often overlooked phase in which residual microorganisms may remain viable and later germinate on dried bamboo slips, thereby promoting further biodeterioration. Notably, Actinomycetes displayed a pronounced capacity to persist under both waterlogged and dried conditions (14.7% in waterlogged S12; 12.9% in dried JD2 and 26.1% in JD3), underscoring their resilience and adaptability across a wide range of moisture content. This dual persistence indicates that Actinomycetes constituted key agents of biodegradation in both waterlogged and dried bamboo slips. Although our results demonstrate that the drying stage functions as an effective environmental filter and that only a limited number of OTUs are shared across the three stages, Actinomycetes appeared to act as broad-spectrum degraders in archeological bamboo slips. This ecological versatility is likely attributable to their ability to tolerate anoxic or hypoxic conditions, as well as their capacity to secrete cellulolytic enzymes (for example, *Pseudonocardia* detected in S12) that facilitate cellulose degradation, despite their frequent isolation from mural paintings rather than waterlogged substrates. In addition, many actinomycetes are known to produce antibiotics or other antimicrobial compounds, which can inhibit competing bacteria and confer a competitive advantage within complex microbial communities [3,14,42,43].

To investigate the niche-specific fungal degradation mechanisms across the environmental filter imposed by the drying stage, three fungi isolated from water-solid-air surface (*F. minima*), dried bamboo slips (*A. saccharicola*) and box surface sample (*X. pulvereus*) were analyzed. At water-solid-air niche, fungi developed specialized traits that enable them to become dominant. This oxygen-rich transitional niche favors the early establishment of dominant fungi compared with the fully waterlogged environment [44,45,46]. Microstructural observations by SEM showed densely distributed and intertwined hyphal structures forming a network-like morphology on sample S11. The most abundant microorganism within this network was successfully isolated and identified as *F. minima* (Figure 7A). Furthermore, FTIR and Py-GC/MS analyses confirmed that *F. minima* was capable of producing melanin, a pigment widely recognized for its role in enhancing fungal tolerance to environmental stresses [47,48,49]. In the context of the water-solid-air interface, melanin production may contribute to persistence under fluctuating moisture and oxidative conditions. Taken together, the dense hyphal organization observed by SEM and the presence of melanin-related chemical signatures are consistent with a biofilm-like colonization strategy, although preparation-related artifacts of SEM cannot be fully excluded. In fungal systems, melanized biofilm-like growth is commonly associated with improved resistance to desiccation, oxidative stress, and ultraviolet radiation [42,43,44,50,51,52,53]. A parallel can be drawn with the Lascaux Cave, where quicklime and benzalkonium chloride were applied to control *Fusarium solani*. However, the treatment ultimately failed due to biofilm-mediated protection and the degradation of benzalkonium chloride by *Pseudomonas fluorescens*. Moreover, inappropriate biocide application promoted the proliferation of other melanized fungi, including *Verticillium* sp. and *Scolecobasidium* sp., both of which were identified as melanized taxa. Melanized fungi generally exhibited reduced susceptibility to biocides, rendering them among the most difficult microorganisms to eradicate [54,55,56]. These outcomes highlight how improper biocide use reflects a neglect of ecological principles in cultural heritage conservation, particularly when melanized fungi are involved [57].

In the oxygen-rich niche of the dried bamboo slips, *A. saccharicola* isolated from the dried materials plays an active role in degradation of bamboo slips. It has been reported that the genus *Apiospora* exhibits a well-established bambusicolous (bamboo-inhabiting) ecology [21]. The pronounced hemicellulolytic activity of *A. saccharicola* supports its role as a primary agent of bamboo biodegradation. In the oligotrophic environment characterized by low water activity on the storage box surface, the xerophilic fungus *X. pulvereus*, emerged as the dominant taxon, representing a potential future risk. This observation indicates that microbial threats to bamboo slips may persist even after drying and subsequent storage [58].

Our study provides a basis for developing stage-specific preservation strategies for organic cultural heritage. The Changsha Zoumalou Bamboo Slips were excavated from an ancient well, and long-term waterlogged storage was indispensable for maintaining their original condition and preventing fungal colonization. In most museums, the soaking water for waterlogged bamboo slips is routinely replaced according to temperature and conservation status. However, the effectiveness of water replacement depends strongly on the preservation state of the artifacts. In this study, the bamboo slips included both fully submerged waterlogged slips (S10, S12) and partially exposed, semi-waterlogged slips (S11). The majority of waterlogged bamboo slips were fully submerged (e.g., S10 and S12), and thus their microbial deterioration patterns are representative of most waterlogged bamboo and wooden artifacts. The important contributors to biodeterioration are rotifers and protozoa, together with bacteria affiliated with Pseudomonadota and Actinomycetota. Rotifers and Protozoa depended strongly on aquatic conditions for survival and activity and can therefore be effectively reduced through water replacement. In contrast, Actinomycetota are more difficult to eliminate because of their broad cellulolytic capacity and facultative anaerobic metabolism. For semi-waterlogged bamboo slips such as S11, the conservation challenge is considerably greater. Dense surface-associated fungal growth, potentially exhibiting biofilm-like characteristics, together with the high stress tolerance of fungal spores, may limit the effectiveness of routine water replacement. In the absence of appropriate antimicrobial measures, the drying stage applied at later stages could therefore promote more pronounced fungal proliferation. Moreover, microorganisms persisting on waterlogged bamboo slips may act as biological reservoirs for subsequent colonization of dried bamboo slips if antimicrobial interventions are not implemented before or during the drying process.

## 5. Conclusions

In conclusion, this study demonstrates that the drying process functions as an environmental filter that selectively suppresses aquatic microorganisms while favoring taxa adapted to low water availability. At the same time, the persistence of a subset of prokaryotic taxa across waterlogged and dried slips indicates that microorganisms present during the waterlogged phase may act as residual biological sources for subsequent colonization after drying. In this context, Actinomycetes represent a particularly relevant risk group, whose broad ecological tolerance, as documented in previous studies, provides a plausible explanation for their persistence across preservation stages. Furthermore, to elucidate the functional basis of this ecological filtering, we demonstrate that dominant fungal taxa are structured along key environmental gradients, including oxygen availability, water activity, and nutrient accessibility. *F. minima* occupy the water-solid-air interface likely, relying on biofilm-like surface colonization and melanin production to withstand physicochemical stress. *A. saccharicola* prevail on dried bamboo slips, exploiting oxygen-rich conditions through active polysaccharide degradation. The xerophilic fungi *X. pulvereus* persist on storage surfaces under oligotrophic and low-water-activity conditions. Accordingly, effective preservation of organic cultural heritage should prioritize stage-specific interventions aligned with dominant functional traits rather than taxonomic identity alone.

## Figures and Tables

**Table 1:** Assignment of the main absorbance bands in the infrared spectra (FTIR) obtained from *F. minima* extracts.

Wavenumber (cm^−1^)	Assignment	References
3300–3500	*ν*O-H; *ν*N-H	[25,26,27]
2926, 2850	Aliphatic *ν*C-H2 and *ν*C-H3	[25,27,28]
1648–1653	*ν*C=O; *ν*C=C aromatic (from conjugated quinone structures)	[26,28,29]
1532	*δ*N-H; *ν*C-N	[26,27,28,29]
1411–1415	*ν*C-N; *δ*C-H3 and *δ*C-H2	[25,26,27,28,29]
1241	*ν*C-OH (alcoholic, carboxylic, phenolic groups)	[25,27,28,29]
1049–1051	Alcoholic C-O; C-H in-plane of aliphatic structure	[25,26,27,28,29]

**Table 2:** Main pyrolysis products of melanin and their annotations based on the NIST database.

Peak Number	Time (min)	Annotation Results	Peak Area (%)
1	1.65	2-methyl-Furan	6.39
2	3.36	Toluene	6.87
3	4.05	Pyrrole	0.57
4	5.77	Ethylbenzene	1.4
5	11.21	1-Undecene	4.45
6	12.80	1-Dodecene	6.16
7	14.08	1-Tridecene1	4.84
8	15.15	1-Tetradecene1	9.2
9	15.36	Indole	2.89
10	16.12	1-Pentadecene	5.09
11	16.16	Pentadecane	4.6
12	16.68	2,4-Di-tert-butylphenol	8.46
13	17.01	Cetene	4.01
14	17.74	8-Heptadecene	2.57
15	17.85	1-Heptadecene	1.73
16	17.88	Heptadecane	2.89
17	18.63	1-Nonadecene	1.58
18	19.10	1-(4-Methyl-3-nitrophenyl) pyrrole-2,5-dione	4.97
19	19.73	Hexadecanoic acid, methyl ester	2.97
20	21.09	Methyl stearate	7.05
21	21.83	Hexadecanamide	5.7
22	22.97	cis-9-Octadecenoamide	2.43
23	23.06	Octadecanamide	2.68
24	24.13	Pyrrolidine, 1-(1-oxooctadecyl)-	0.51

## Data Availability

The original contributions presented in this study are included in the article/Supplementary Materials. Further inquiries can be directed to the corresponding author.

## Supplementary Materials

The following supporting information can be downloaded at: https://www.mdpi.com/article/10.3390/jof12010066/s1, Figure S1: Three-dimensional hyphal network on bamboo slip sample S11 at the water-solid-air interface revealed by multi-scale SEM imaging; Figure S2: Maximum likelihood tree based on ITS genes. Seiridium phylicae was used as the outgroup; Figure S3: The annotation of 18S rRNA gene ASV1 and ASV2; Figure S4: Maximum likelihood phylogenetic tree of fungal ITS sequences from TA cloning of sample JD1; Figure S5: Maximum likelihood phylogenetic tree of fungal ITS sequences from TA cloning of sample JD2; Figure S6: Maximum likelihood phylogenetic tree of fungal ITS sequences from TA cloning of sample JD9; Figure S7. Sampling of S11 sample, streaking of the sampling swab onto the initial agar plates (including LB and PDA plates), apparent morphology observed after incubating the initial plates at 28 °C for 8 days, and observation of the Fonsecaea minima colonies.
