# Supplementary material for: Environmental Filtering Drives Microbial Community Shifts and Functional Niche Differentiation of Fungi in Waterlogged and Dried Archeological Bamboo Slips
Source: J Fungi (Basel). 2026 Jan 14;12(1):66. doi: 10.3390/jof12010066 (PMC12843188; doi:10.3390/jof12010066)
Supplement: Supplementary file 1 [file jof-12-00066-s001.zip › jof-4003581-supplementary.pdf]

Environmental Filtering Drives Microbial Community Shifts and Functional Niche Differentiation of Fungi in Waterlogged and Dried Archaeological Bamboo Slips

Liwen Zhong [1,2], Weijun Li[3], Guoming Gao [1,2], Yu Wang[1,2], Cen Wang[1,2] and Jiao Pan [1,2]*

1 Key Laboratory of Archaeomaterials and Conservation, Ministry of Education, University of Science and Technology Beijing, Beijing, 100083, China; zhongleven@outlook.com (L.Z.); m202411471@xs.ustb.edu.cn (G. G.); d202310766@xs.ustb.edu.cn (Y.W.); D202410811@xs.ustb.edu.cn (C.W.).

2 Institute for Cultural Heritage and History of Science & Technology, University of Science and Technology Beijing, Beijing 100083, China

3 Changsha Jiandu Museum, Changsha, Hunan, 410002, China; liweijun0119@foxmail.com (W.L.)

* Correspondence: jiaopan@ustb.edu.cn; Tel.: +86-138-2006-8355

Figure S1. Three-dimensional hyphal network on bamboo slip sample S11 at the water-solid-air interface revealed by multi-scale SEM imaging.

Figure S2. Maximum likelihood tree based on ITS genes. *Seiridium phylicae* was used as the outgroup.

Figure S3. The annotation of 18S rRNA gene ASV1 and ASV2.

Figure S4. Maximum likelihood phylogenetic tree of fungal ITS sequences from TA cloning of sample JD1.

Figure S5. Maximum likelihood phylogenetic tree of fungal ITS sequences from TA cloning of sample JD2.

Figure S6. Maximum likelihood phylogenetic tree of fungal ITS sequences from TA cloning of sample JD9.

Figure S7. Sampling of S11 sample, streaking of the sampling swab onto the initial agar plates (including LB and PDA plates), apparent morphology observed after incubating the initial plates at 28 °C for 8 days, and observation of the *Fonsecaea minima* colonies.

S11a

S11b

S11c

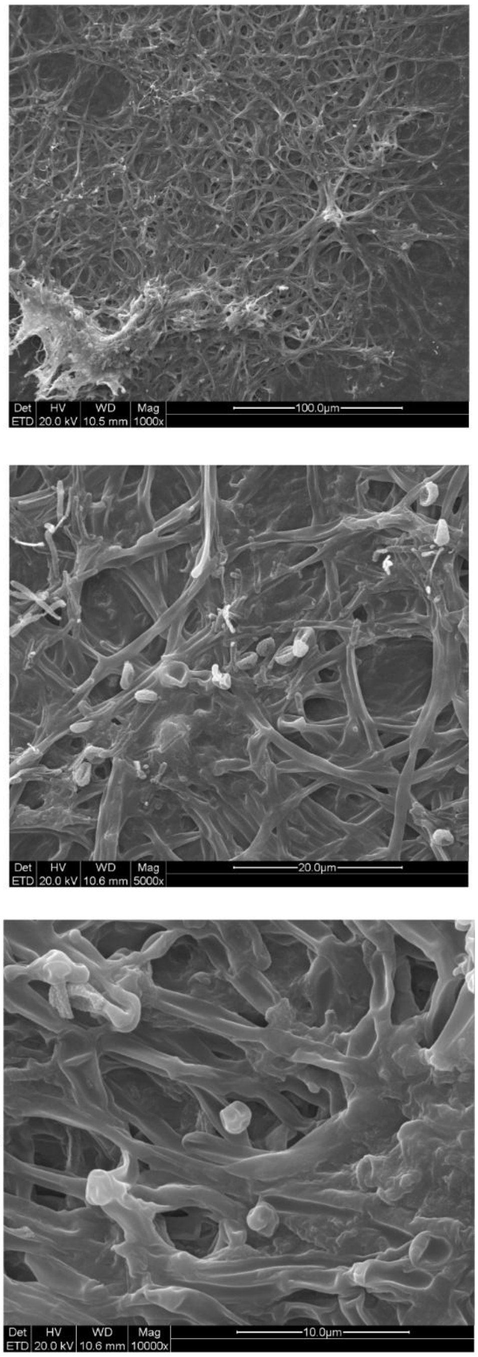

Figure S1. Multi-scale SEM imaging of hyphal structures on bamboo slip sample S11 at the water-solid-air interface.

(S11a) Low-magnification view (1,000 ×) showing the overall extent and interconnectivity of filamentous structures on the sample surface.

(S11b) Intermediate-magnification view (5,000 ×) highlighting dense intertwining and overlapping of hyphae.

(S11c) High-magnification view (10,000 ×) allowing for the identification of fungal hyphae based on key ultrastructural features.

In the water-solid-air sample S11 (Figure 2, Sample S11), SEM imaging at progressively higher magnifications was used to examine the apparent architecture of the microbial assemblage. At low magnification (1,000 ×), an extensive and interconnected network of filamentous structures was observed covering the bamboo surface (Figure 2, Sample S11). Intermediate magnification (5,000 ×) revealed dense intertwining and overlapping of filaments, which may suggest of a potential three-dimensional organization (Figure S6). Finally, high-magnification imaging (10,000 ×) enabled the identification of these filaments as fungal hyphae, characterized by smooth cell walls, natural branching points, and a rounded, wrinkled terminus (Figure. S6). While the overall morphology suggests a three-dimensional network, the perceived spatial arrangement could still be influenced by sample preparation or imaging perspective.

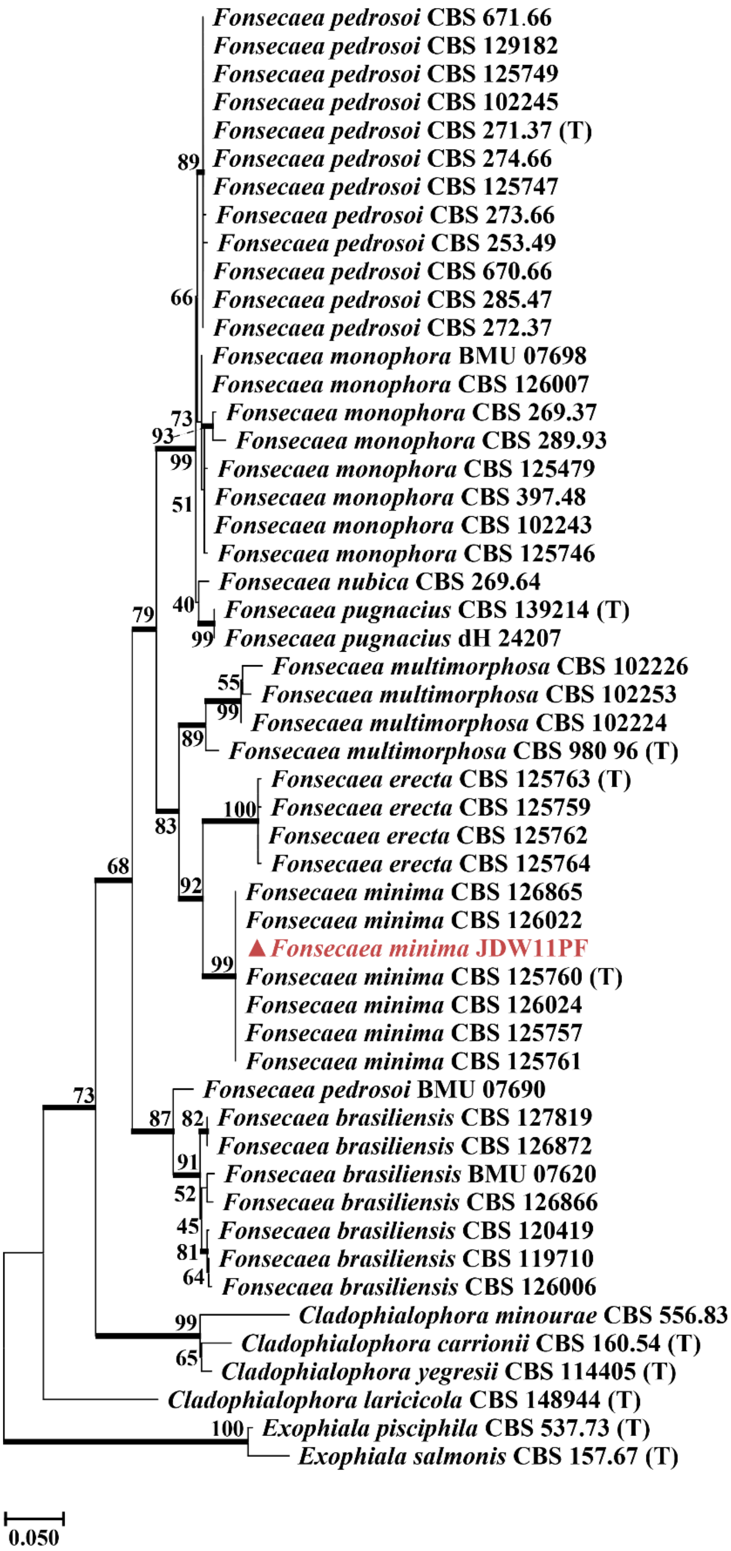

Figure S2. Maximum likelihood tree based on ITS genes. *Seiridium phylicae* was used as the outgroup.

Cladophialophora laricicola, *Cladophialophora carrionii*, *Cladophialophora yegresii*, *Cladophialophora minourae*, *Exophiala salmonis* and *Exophiala pisciphila* were used as the outgroup. (T) = type

strain of the species. Bootstrap support was calculated from 1000 replicates; values > 70% are indicated in bold on the branches.

The ITS-based maximum likelihood tree, comprising a total of 52 sequences, placed strain *F. minima* JDW11PF within the *F. minima* clade with high support (99%; Figure S3). This analysis supplements the concatenated tree in Figure 4 by encompassing all currently recognized species in the genus *Fonsecaea*, thus expanding the taxonomic coverage presented.

ASV1 Description	Scientific Name	Max Score	Total Score	Query Cover	E value	Per. Ident	Acc. Len	Accession
Exophiala nigra CBS 535.94 18S rRNA gene, partial sequence; from TYPE material	Exophiala nigra	564	564	100%	3e-156	100.00%	1762	NG_062119.1
Phaeoannellomyces elegans 18S rRNA gene, exon 1 and 2	Phaeoannellomy…	564	564	100%	3e-156	100.00%	1756	X80708.1
N.nigra 18S rRNA gene, exons 1 and 2	Exophiala nigra	564	564	100%	3e-156	100.00%	1752	X80706.1
Exophiala sideris CBS 121818 18S rRNA gene, partial sequence; from TYPE material	Exophiala sideris	564	564	100%	3e-156	100.00%	1749	NG_062072.1
Exophiala sideris voucher CBS:121818 small subunit ribosomal RNA gene, partial sequence	Exophiala sideris	564	564	100%	3e-156	100.00%	1749	HQ441174.1
Exophiala sp. SST-2011 voucher CBS:127096 small subunit ribosomal RNA gene, partial sequence	Exophiala sideris	564	564	100%	3e-156	100.00%	1744	HQ441175.1
Exophiala sp. SL-2015c culture-collection CGMCC:3.17345 18S ribosomal RNA gene, partial sequence	Exophiala sp. SL…	564	564	100%	3e-156	100.00%	914	KP347966.1
Chaetothyriales sp. TRN14 isolate TRN 14 small subunit ribosomal RNA gene, partial sequence	Chaetothyriales …	564	564	100%	3e-156	100.00%	1938	FJ358321.1
Chaetothyriales sp. TRN493 isolate TRN 493 small subunit ribosomal RNA gene, partial sequence	Chaetothyriales …	564	564	100%	3e-156	100.00%	2027	FJ358331.1
Chaetothyriales sp. S10 partial 18S rRNA gene, strain S10	Chaetothyriales …	564	564	100%	3e-156	100.00%	1769	LT220851.1
Exophiala wilsonii CCF 5674 18S rRNA gene, partial sequence; from TYPE material	Exophiala wilsonii	558	558	100%	2e-154	99.67%	1670	NG_243003.1
Fonsecaea monophora strain BMU 07698 18S ribosomal RNA gene, partial sequence	Fonsecaea mon…	558	558	100%	2e-154	99.67%	1380	KM658052.1

ASV2 Description	Scientific Name	Max Score	Total Score	Query Cover	E value	Per. Ident	Acc. Len	Accession
Capronia sp. WUC 15.551 small subunit ribosomal RNA gene, partial sequence	Capronia sp. W…	564	564	100%	3e-156	100.00%	1599	FJ358295.1
Uncultured fungus CL25-1-F gene for 18S rRNA, partial sequence	uncultured fungus	564	564	100%	3e-156	100.00%	673	LC365199.1
Herpotrichiellaceae sp. F-6 18S ribosomal RNA gene, partial sequence	Herpotrichiellac…	564	564	100%	3e-156	100.00%	1787	EU090194.1
Exophiala sp. DMKU:MC01 gene for 18S ribosomal RNA, partial sequence	Exophiala sp. D…	558	558	100%	2e-154	99.67%	1000	LC799028.1
Exophiala nigra CBS 535.94 18S rRNA gene, partial sequence; from TYPE material	Exophiala nigra	558	558	100%	2e-154	99.67%	1762	NG_062119.1
Phaeoannellomyces elegans 18S rRNA gene, exon 1 and 2	Phaeoannellom…	558	558	100%	2e-154	99.67%	1756	X80708.1
N.nigra 18S rRNA gene, exons 1 and 2	Exophiala nigra	558	558	100%	2e-154	99.67%	1752	X80706.1
Exophiala sideris CBS 121818 18S rRNA gene, partial sequence; from TYPE material	Exophiala sideris	558	558	100%	2e-154	99.67%	1749	NG_062072.1
Exophiala alcalophila CBS 520.82 18S rRNA gene, partial sequence; from TYPE material	Exophiala alcalo…	558	558	100%	2e-154	99.67%	1741	NG_062074.1
Exophiala sp. isolate KUNCC24-17778 small subunit ribosomal RNA gene, partial sequence	Exophiala sp.	558	558	100%	2e-154	99.67%	901	PP869235.1
Exophiala sideris voucher CBS:121818 small subunit ribosomal RNA gene, partial sequence	Exophiala sideris	558	558	100%	2e-154	99.67%	1749	HQ441174.1
Exophiala sp. SST-2011 voucher CBS:127096 small subunit ribosomal RNA gene, partial sequence	Exophiala sideris	558	558	100%	2e-154	99.67%	1744	HQ441175.1
Exophiala sp. SL-2015c culture-collection CGMCC:3.17345 18S ribosomal RNA gene, partial sequence	Exophiala sp. S…	558	558	100%	2e-154	99.67%	914	KP347966.1
Exophiala sp. strain FHG001150 small subunit ribosomal RNA gene, partial sequence	Exophiala sp.	558	558	100%	2e-154	99.67%	849	OL823165.1

Figure S3. The annotation of 18S rRNA gene ASV1 and ASV2 of Sample 11.

The results of BlAST, which showed annotation of ASV1 and ASV2, indicate ASV1 and ASV2 can identified as Herpotrichiellaceae species, due to the low resolution of 18S rRNA gene.

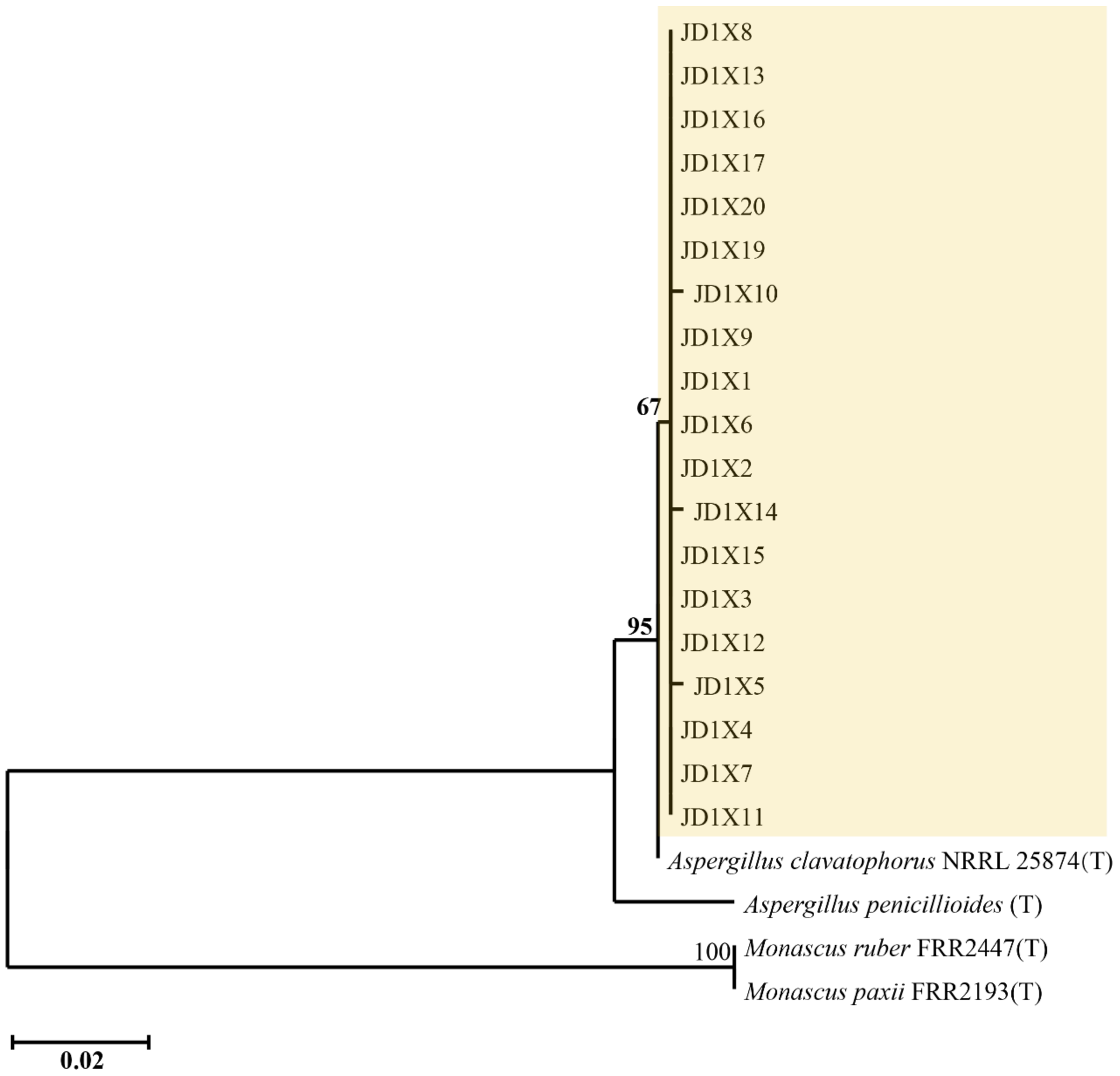

Figure S4. Maximum likelihood phylogenetic tree of fungal ITS sequences from TA cloning of sample JD1.

The tree was rooted with *Monascus ruber*. Bootstrap values (> 50%) from 1000 replicates are shown at the nodes. (T) = type strain of the species or of one of its synonyms.

The tree illustrates the phylogenetic placement of 19 TA clones obtained from the sample. Eighteen clones form a tight clade with the reference sequence of *Aspergillus clavatophorus* and *Aspergillus penicillioides*. It demonstrates that all 19 clones are identified as *Aspergillus clavatophorus* or *Aspergillus penicillioides*.

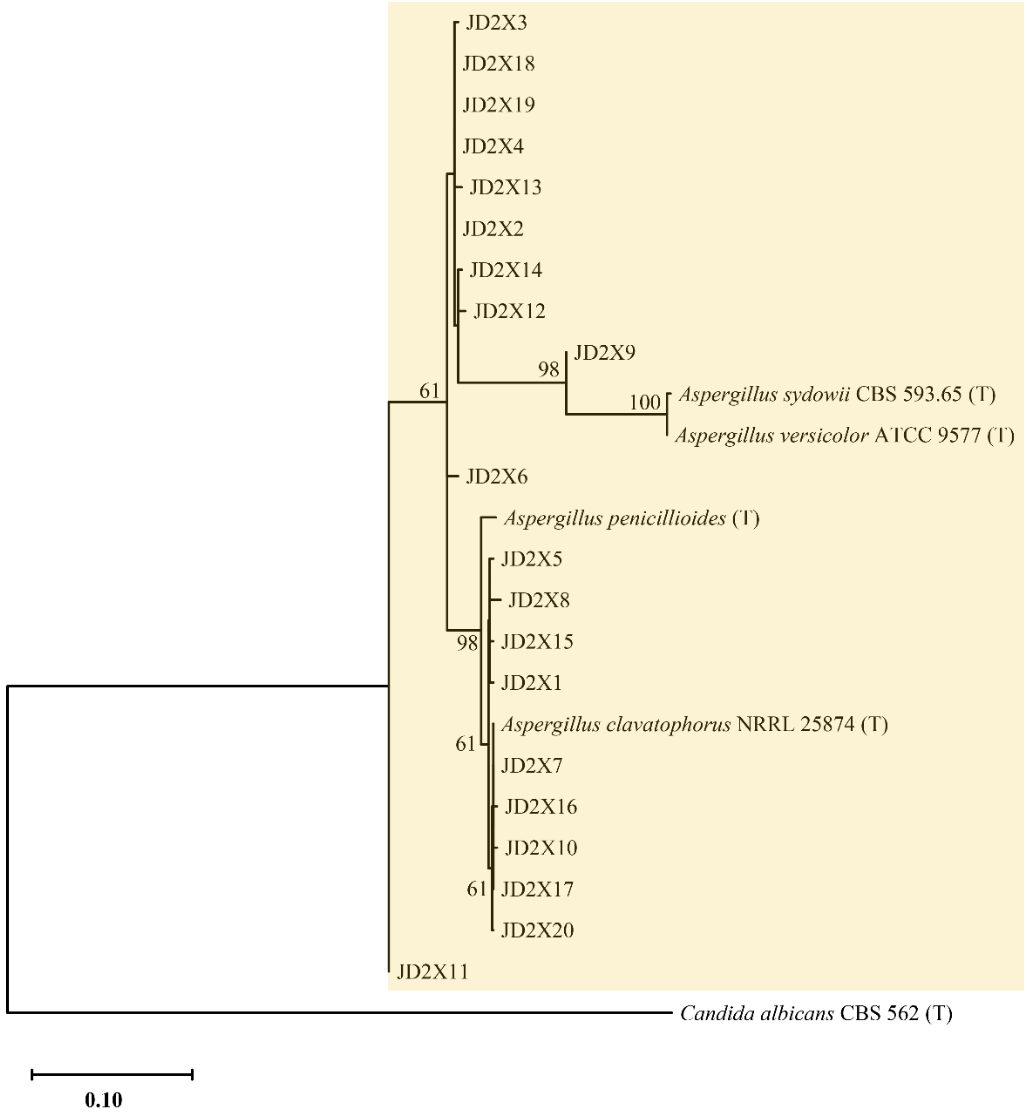

Figure S5. Maximum likelihood phylogenetic tree of fungal ITS sequences from TA cloning of sample JD2.

The tree was rooted with *Candida albicans*. Bootstrap values (> 50%) from 1000 replicates are shown at the nodes. (T) = type strain of the species or of one of its synonyms.

The tree illustrates the phylogenetic placement of 20 TA clones obtained from the sample. 20 clones form a tight clade with the reference sequence of *Aspergillus clavatophorus*, *Aspergillus penicillioides*, *Aspergillus versicolor* and *Aspergillus sydowii*. It indicates that most clones belong to *Aspergillus* genus except JD2X11 and JD2X6.

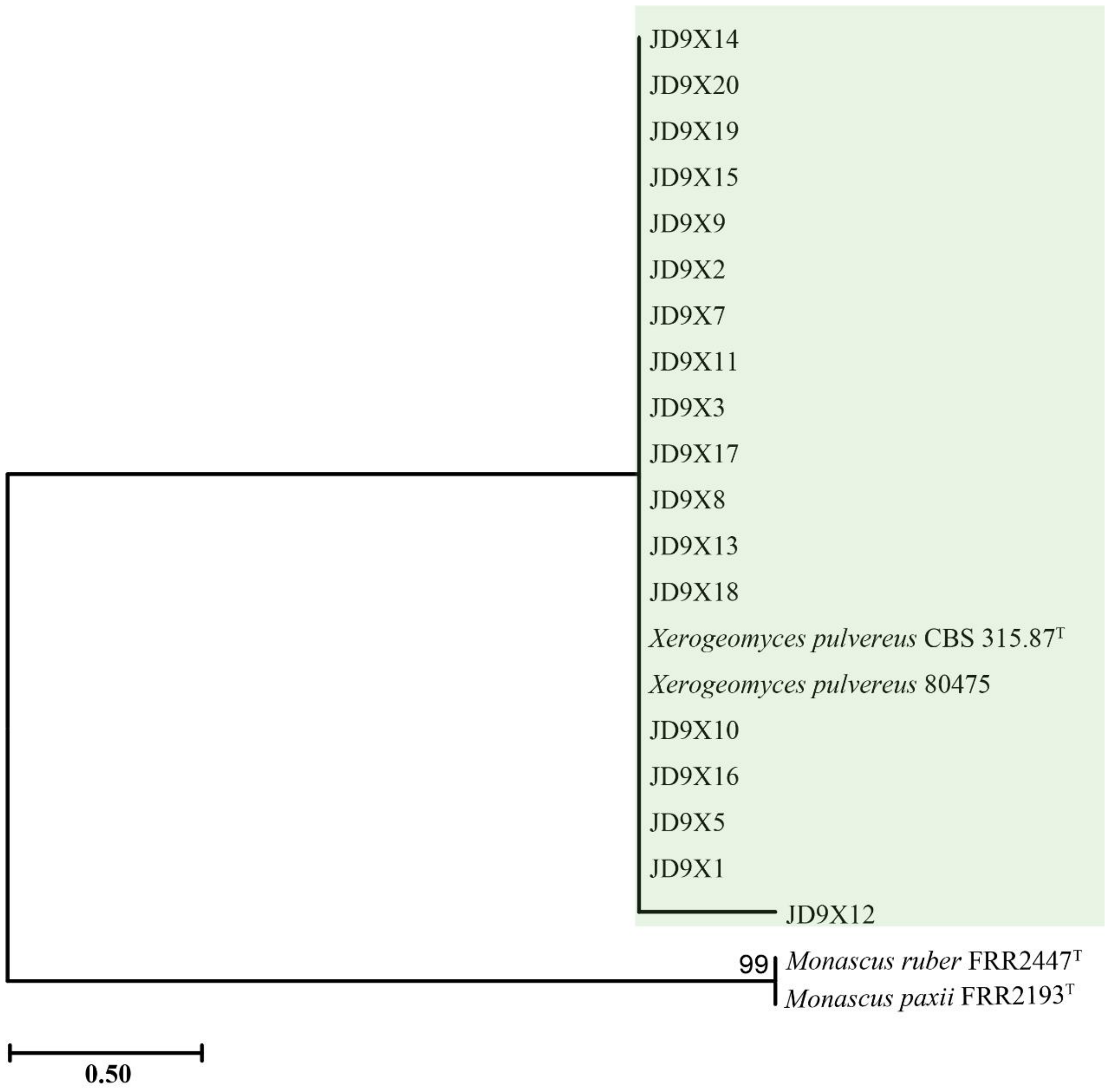

Figure S6. Maximum likelihood phylogenetic tree of fungal ITS sequences from TA cloning of sample JD9.

The tree illustrates the phylogenetic placement of 18 TA clones obtained from the sample. The tree was rooted with *Monascus ruber*. Bootstrap values (> 50%) from 1000 replicates are shown at the nodes. (T) = type strain of the species or of one of its synonyms.

The analysis includes reference sequences of closely related xerophilic fungi. Eighteen clones form a tight clade with the reference sequence of *Xerogeomyces pulvereus* [53], with no internal branch structure, indicating identical or nearly identical ITS sequences. This study identified the isolated strain as *X. pulvereus* through phylogenetic analysis of the ITS sequence. This identification is strongly supported by the latest record from the authoritative taxonomic database Index Fungorum (Minnis & Lindner, 2023). The record indicates that *Xerogeomyces* is a newly established genus in 2023, with *X. pulvereus* as its type species [18].

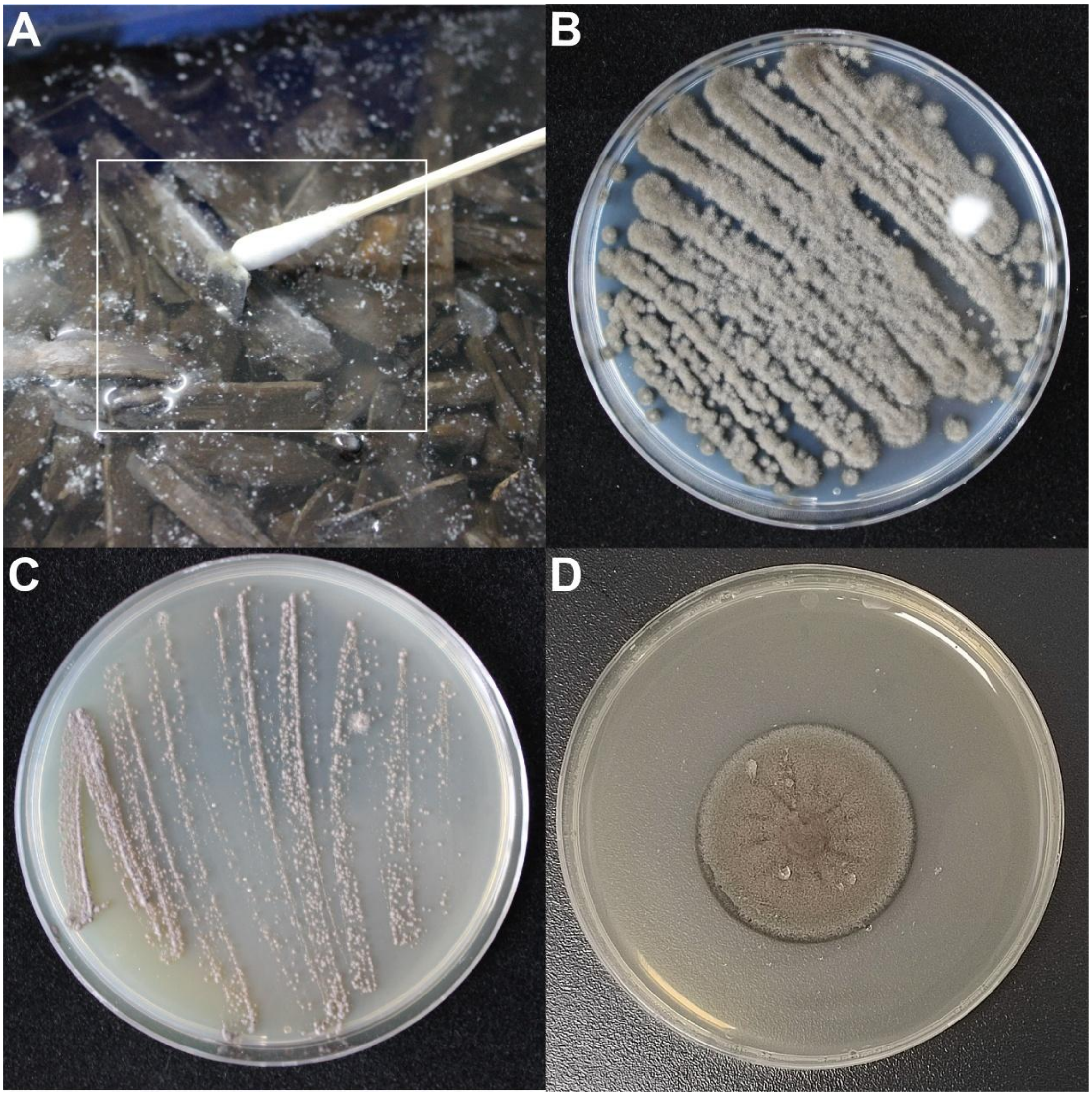

Figure S7. Sampling of S11 sample, streaking of the sampling swab onto the initial agar plates (including LB and PDA plates), apparent morphology observed after incubating the initial plates at 28 °C for 8 days, and observation of the *Fonsecaea minima* colonies.

The picture shows the coordinates among the appearances of the sampling site, initial agar plates and the *F. minima,* which have greenish black in color.
